# IDAF: Iterative Dual-Scale Attentional Fusion Network for Automatic Modulation Recognition

**DOI:** 10.3390/s23198134

**Published:** 2023-09-28

**Authors:** Bohan Liu, Ruixing Ge, Yuxuan Zhu, Bolin Zhang, Xiaokai Zhang, Yanfei Bao

**Affiliations:** 1 Institute of Systems Engineering, Academy of Military Science of the People’s Liberation Army, Beijing 100083, China; 2 National Key Laboratory of Science and Technology on Communication, University of Electronic Science and Technology of China, Chengdu 611731, China; 3 College of Communications and Engineering, Army Engineering University of PLA, Nanjing 210007, China

**Keywords:** automatic modulation recognition, multimodal learning, convolutional neural network, attention mechanism

## Abstract

Recently, deep learning models have been widely applied to modulation recognition, and they have become a hot topic due to their excellent end-to-end learning capabilities. However, current methods are mostly based on uni-modal inputs, which suffer from incomplete information and local optimization. To complement the advantages of different modalities, we focus on the multi-modal fusion method. Therefore, we introduce an iterative dual-scale attentional fusion (iDAF) method to integrate multimodal data. Firstly, two feature maps with different receptive field sizes are constructed using local and global embedding layers. Secondly, the feature inputs are iterated into the iterative dual-channel attention module (iDCAM), where the two branches capture the details of high-level features and the global weights of each modal channel, respectively. The iDAF not only extracts the recognition characteristics of each of the specific domains, but also complements the strengths of different modalities to obtain a fruitful view. Our iDAF achieves a recognition accuracy of 93.5% at 10 dB and 0.6232 at full signal-to-noise ratio (SNR). The comparative experiments and ablation studies effectively demonstrate the effectiveness and superiority of the iDAF.

## 1. Introduction

Automatic modulation recognition (AMR) [1,2] is the process of identifying the modulation of the received signal in the absence of sufficient a priori information. Defining the modulation is necessary for correct demodulation, which is fundamental in spectrum monitoring [3], information countermeasures [4], cognitive radio [5], etc. With the increasing development of wireless communication technology, the modulation of signals tends to be diversified, and the number of frequency-using devices is increasing. Therefore, the study of real-time and efficient AMR is of great practical significance.

The mainstream AMR methods are divided into two categories, i.e., likelihood theory-based (LB-AMR) [1,6,7] and the feature-based (FB-AMR) [2,8] methods. However, the performance of these traditional methods relies on manually estimated parameters [9], which leads to harder feature extraction under the high data transmission rates [10]. Instead of relying on artificial derivation to extract features, deep learning models feed signals directly into the network for end-to-end learning. Experiments have confirmed that the methods based on deep learning (DL) have better recognition accuracy than the traditional LB-AMR and FB-AMR methods [11]. At present, a large number of deep neural networks [12] such as Convolutional Neural Network (CNN) [13], Denoising Automatic Encoder (DAE) [14], and Recurrent Neural Network (RNN) [15] are all introduced into AMR tasks. In the existing DL-AMR methods, most take a single modality as the input data type, such as in-phase/quadrature (I/Q) [15], amplitude/phase series (A/P) [16], the Welch spectrum, square spectrum, and the fourth power spectrum [17,18]. However, a single modality only contains the limited identifying information that is required for recognition completely from specific domains.

For DL-AMR methods [13,14,15], different input data types have their own advantages. As shown in Table 1, input data from different modalities perform distinctively well for particular modulations due to the domain gap. Obviously, the I/Q, A/P, and spectral data have significant distinguishing abilities for the PAM, QAM, and PSK modulations, respectively. However, the use of single-domain data formats does not provide a sufficiently efficient or complete view for recognition, which is due to the fact that different modes contain specific properties. In the actual complex and changing electromagnetic environment [19,20], lacking sufficient samples and a priori information, it is critical to make the best possible utilization of the limited spectrum resources for identification purposes. Converting signals into multimodal data enables unique information to be obtained from different domains, and meanwhile maximizing the available information content.

In recent years, several studies have also focused on the advantages of multimodal information fusion for AMR tasks. In [24], modality discriminative features are captured separately using three Resnet networks, and I/Q, A/P, and the amplitudes of spectrum, square spectrum, and fourth power spectrum features are concatenated with the corresponding bitwise summation. The authors in [25] propose a dual-stream structure based on CNN-LSTM (DSCLDNN), which combines the characteristics of I/Q with A/P via pairwise cross-interaction of the characteristics of the two streams. Specifically, the DSCLDNN multiplies I/Q and A/P features with an outer product. Unlike the above direct addition or multiplication fusion approach, Ref. [23] uses a PNN (Product-based Neural Network) model to cross-fuse the three modal features in a fixed order. However, most of the above methods fuse multimodal features via direct or crosswise summation or outer product, which tends to ignore the variability of different modes and their different impacts on modulation identification.

Generally, the attention mechanism [26,27] can identify the channel-wise importance. Therefore, each modality has adaptively obtained its respective attention weight. For a feature map, attention weights need to be focused on both the channel and spatial dimensions. Channel attention such as SENets (Squeeze-and-Excitation Networks) [26], GSoPNet (Global Second-Order Pooling Convolutional Networks) [28], and SRM (Style-Based Recalibration Module) [29] extract the attention information of different channels to distribute greater weight to important channels. For the spatial dimension, the attention mechanisms such as GENet (Gather and Excite Network) [27], RAM (Recurrent Attention Model) [30], and self-attention [31] are used to extract important spatial regions or spatial locations of high relevance.

For the existing dual-channel attentional fusion methods, their characteristics were discovered in terms of the fusion approach. From Table 2, it can be seen that existing two-channel attention fusion can be broadly classified into two types: (1) Direct aggregation. Extract the global attention weights and then multiply them linearly with the global/local feature maps, and finally, connect with the original feature maps through a simple short jump. (2) Gating unit. Assigns importance to each modal feature simultaneously via multimodal gating units. (3) Balanced weighting. Constrain the sum of the weights of the feature maps to 1 and fully interact with the different scale information of the context through long and short jump connections. In our iDAF, the unified framework of soft selection is condensed and iterated. For multi-channel inputs composed of multimodal signals, the structures of the channel and spatial attention mechanisms were borrowed for the dual-scale attentional fusion (DAF) that we designed. Specifically, the dual channels are local and global branches. On the local branch, the spatial attention mechanism extracts local high-level feature details, while the channel attention mechanism on the global branch assigns attention weights to the different modal channels.

The main contribution of this work can be summarized as follows:

- We propose a deep learning method based on iterative dual-scale attentional fusion (iDAF), which complements the properties and complementarity of multimodal information with each other to achieve better recognition.
- We design two embedding layers to extract the local and global information, extracting information that promotes recognition from different-sized respective fields. The extracted features are sent into the iterative dual-channel attention module (iDCAM), which consists of the local and global branch. The branches respectively focus on the details of the high-level features and the variability across modalities.
- Experiments on the RML2016.10A dataset demonstrate the validity and rationalization of iDAF. The highest accuracy amount of 93.5% is achieved at 10 dB and the recognition accuracy is 0.6232 at full SNR.

## 2. Related works

### 2.1. Research on Traditional AMR Methods

For the AMR task, two traditional methods are LB-AMR and FB-AMR. The LB-AMR typically uses probability theory and hypothesis testing theory, while the FB-AMR is achieved by selecting representative features that best reflect the differences between the modulated signals. LB-AMR mainly includes the average likelihood ratio test [2,36], the generalized likelihood ratio test [37], and the mixed likelihood ratio test [38]. However, although the likelihood technique is optimal in the sense of minimizing the probability of misclassification, the practical implementation is affected by computational complexity [16] and it is difficult to determine the appropriate analytic solution to the decision function [2]. In contrast, FB-AMR has a low computational cost and it achieves near-optimal performance, which has proven the validity of extracted features through mathematical calculation [22,39,40]. However, the performance of the traditional algorithm relies on manually estimated parameters [9], and it becomes increasingly difficult to extract features with the development of high data transmission rates [10]. Therefore, the strong automatic learning capability of DL models is widely used to accomplish the AMR task.

### 2.2. Study of Different Inputs and DL-Models

DL-AMR methods achieve a high recognition accuracy with different input modalities. By analyzing the I/Q vector [13], based on a simple Convolutional Neural Network (CNN) it achieves a higher accuracy in full SNR than do traditional methods. In [10], the feedforward deep neural network (DNN) was used for pre-training, and the I and Q components were passed through an independent automatic encoder to realize unsupervised feature learning. OFDM signals were converted into I/Q samples in [9], and frequency domain analysis (FDA) pretreatment and l2 regularization were used to achieve high classification accuracy under low SNR. In addition to one-dimensional data, two-dimensional visual representations such as time–frequency and constellation images also show strong representation ability. A quarter spectrum diagram (Q-spectrum diagram) representation is proposed in [3], which is used by well-known convolutional neural networks such as VGG-16, AlexNet, and ResNet18, respectively, with a classification accuracy of more than 98% at a high signal-to-noise ratio. Ref. [41] takes a constellation map as the input of the InceptionResnetV2-TA network when the signal-to-noise ratio is 4dB; the recognition rate on three typical signals is 3% higher than with other algorithms. However, current models focus on information in a single domain of time, phase, and frequency, which results in the underutilization of multimodal signal data. Therefore, we consider complementing the advantages of different modalities based on attentional mechanisms, to facilitate a complete view of the signal being obtained.

## 3. The Proposed Method

In this section, we first preprocess the initial data to obtain a three modalities representation. Then, we introduce iterative dual-scale attentional fusion (iDAF), consisting of data embedding layers and the iterative dual-channel attention module (iDCAM).

### 3.1. Data Preprocessing

This paper aims to identify modulation in a single-input single-output radio transmission system (SISO). The receiver transmits signal *s* through transmission channel *h* to obtain the baseband transmission signal.

(1) $$\begin{array}{c} s\left(i\right)=A\left(i\right)e^{j(\omega l+\varphi )}s\left(i\right)+n\left(i\right),i=1,2,3\ldots N, \end{array}$$

where *s* is the complex baseband signal transmitted by the transmitter under some modulation scheme, ω is the frequency offset, φ is the phase offset, *A* is the communication channel gain, *n* is the Additive Gaussian White Noise (AWGN), and *i* represents the *i*-th value received. The purpose of the automatic modulation recognition task is to transmit signals through the baseband of the receiver and to determine the pattern of modulation recognition, which can be classified as a $P(y=C_{K}|s)$ estimation problem for identifying *K* types of radio modulations.

The key to the recognition task is to obtain the effective features of the signal, while the representational ability of the features extracted by a single modality is limited especially in the case of low SNR. In order to cover the identification of the amplitude, phase, and spectrum characteristics that are required for modulation recognition, three modalities are selected to ensure that the required identifying information is included. I/Q and A/P contain instantaneous amplitude, phase, and frequency information as modality one (IQ) and modality two (AP), respectively. The Welch spectrum, square spectrum, and fourth power spectrum selected as the third modality (SA) represent the spectral characteristics of the signal in the frequency domain.

Therefore, prior to input into the neural network, the original signal symbol is transferred to the three modal representations in the following ways:

- In-phase/orthogonal (IQ): Generally, the receiver stores the signal in the modality of I/Q to facilitate mathematical operation and hardware design, which is expressed as follows:
  (2) VIQ=IQ=Re[s(1),s(2),…,s(N)]Im[s(1),s(2),…,s(N)]=Re[1],Re[2],…,Re[n]Im[1],Im[2],…,Im[n]
  where *I* and *Q* represent the in-phase and quadrature components, and Re and Im refer to the real and imaginary parts of the signal, respectively.
- Amplitude/phas (AP): The instantaneous amplitude and phase of the signal are calculated, expressed as:
  (3) VAP=AP=Amplitude(n)=Re2[n]+Im2[n]Phrase(n)=arctanIm[n]Re[n]
  where the values of *n* are 0,1,2,…,N−1.
- Spectrum (SP): The spectrum expresses the change of frequency over time, which is an important discrimination of different modulations. The calculation of the spectrum is expressed as:
  (4) $$\begin{array}{c} V_{SP}=\left|\sum_{i=0}^{N-1}s{\left(i\right)}^{n}e^{-j2\pi ki/N}\right|,k=0,1,2,\ldots ,N \end{array}$$
  where *n* represents the *n*-th power of the spectrum, including 1, 2, and 4, which correspond to the Welch spectrum, square spectrum, and fourth power spectrum. Here, M1 and M2 represent the signal waveform and frequency, and M3 refers to signal time–frequency characteristics. The feature vectors of the three modalities were normalized into (batchsize ×128).

### 3.2. Iterative Dual-Scale Attentional Fusion Fusion (iDAF)

**Figure 1:**
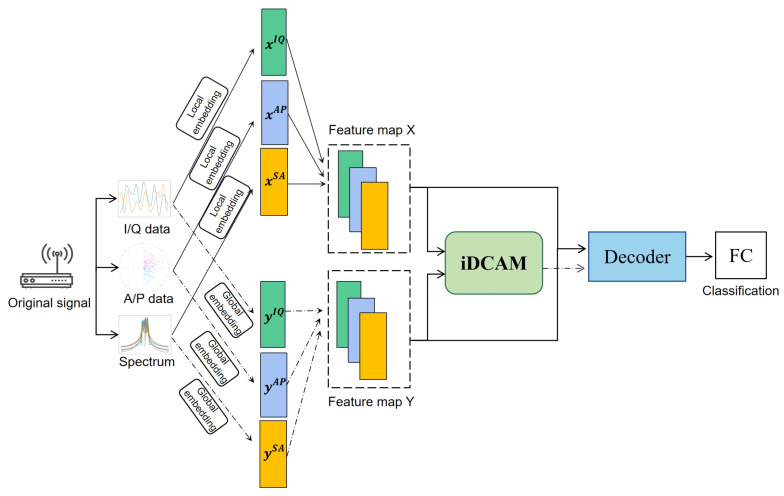
Architecture of the proposed iterative dual-scale attentional fusion (iDAF).

For the iDAF, we designed with two data embedding layers to construct the local and global feature maps, then we sent it into an iDCAM for attention weight assignment, as shown in Figure 1.

#### 3.2.1. Data Embedding

The signal data consist of three modal inputs, including I/Q, A/P, and spectrum analysis. For the original signal, it is preprocessed into three modalities inputs, denoted as $h_{IQ}\in R^{128\times 2}$, $h_{AP}\in R^{128\times 2}$, and $h_{SP}\in R^{128\times 3}$. The preprocessed inputs $h_{m}$ (m∈(IQ,AP,SP)) represent orthogonal information, amplitude-phase domain, and spectral features, respectively.

Due to the variability of the multimodal features, direct fusion would ignore the properties that are unique to different modalities. Therefore, we capture features from both local and global feature maps. The local feature map extracts detailed high-level semantic features, and the global feature map focuses on inter-modal salient characteristics. Therefore, we construct these two feature maps separately using feature extraction networks with different-sized receptive fields.

For the local feature map *X*, the feature extraction network is expected to focus on local details and contextual information. Inspired by CLDNN in [42], we propose the local embedding layer with CNN, LSTM, and DNN, which is fine-tuned to extract local attention information. Firstly, preprocessed data pass through a few convolution layers to model the frequency. Therefore, the long-term features are obtained via undistorted convolution (UD-Conv) layers with channel dimensions of 128, 64, 32, and 16. Concretely, UD-Conv consists of a zero-padding layer of size (2,0,0,0), a convolution layer, the ReLU function, and batch normalization. Using the zero-padding, two columns are added to ensure that the signal features can be transmitted with as little time-frequency information as possible. Following [43], the outputs of CNN are sent into LSTM and DNN. The LSTM layer is a bidirectional recursive model with 100 cells, which makes predictions using information both before and after the current moment in the sequence. The input is passed to the model in the original order, the incoming data in the reverse order, and finally, the forward and reverse outputs are merged. The long-short time series learning capability of the LSTM identifies temporal correlations in the I/Q data with inherent memory properties, and this benefits the learning of the temporal dependencies of instantaneous amplitude and phase [16].

**Figure 2:**
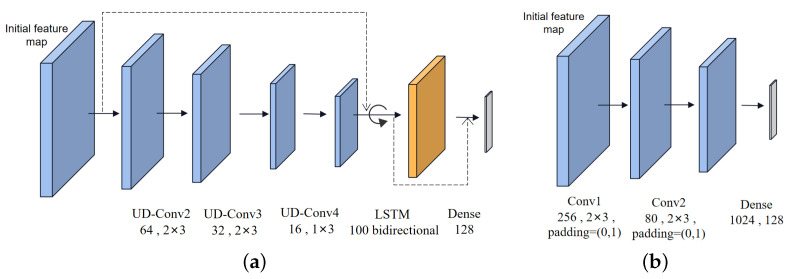
The feature map construction networks. (**a**) The local embedding layer $E_{x}$ for feature map *X*. (**b**) The global embedding layer $E_{y}$ for feature map *Y*.

The residual mapping function is a shortcut path between different layers, which can deepen the communication between deep and shallow neural network features. Inspired by [15], the Resnet has achieved the best performance on classifying signal modulation with a four-convolution-layer structure. After four UD-Conv layers, long-term features are extracted using the convolution layers, while short-term information may be neglected during the convolution process. Therefore, the original data containing the long-short-term features are entered into LSTM, together with the extracted long-term features via the residual connection. Inspired by [44], the extracting capability of CNN is combined with LSTM and DNN. As shown in Figure 2a, the learned short-term features are fed into the dense layer, together with the long-term features previously extracted by CNN. The local embedding layer captures the data characteristics of each modal with unshared parameters, which is expressed as $x_{m}=E_{x}\left(h_{m},\omega_{m}^{E_{x}}\right),\left(m\in \left(IQ,AP,SA\right)\right)$, where $E_{x}$ represents the local embedding layer and $\omega_{m}$ indicates the local network parameters.

To obtain the global feature map *Y*, an optimized CNN with three convolutional layers is utilized to extract features $y_{m}=E_{y}\left(h_{m},\omega_{m}^{E_{y}}\right),\left(m\in \left(IQ,AP,SA\right)\right)$ in the global receptive field in Figure 2b.

#### 3.2.2. Dual-Scale Channel Attention Module

**Figure 3:**
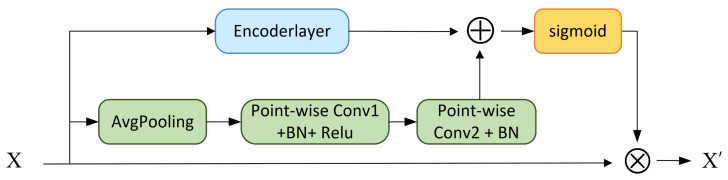
Architecture of the proposed dual-scale channel attention module (DCAM).

After constructing the feature maps in the previous section, the feature maps are fed into an iDCAM. The architecture of the DCAM is a computational unit that can be constructed and superimposed for feature map transformation containing two branches as shown in Figure 3.

The branches include a local attention branch and a global attention branch, correspondingly, for extracting the local identification properties and the channel variability between modalities, respectively. The local attention branch extracts the intra-modal attention through the self-attention mechanism of the Transformer, which extracts the local recognition properties of specific modality features. Meanwhile, the global attention branch increases the receptive field via pooling to obtain inter-modal global attention in the channel dimension. The feature maps are respectively fed into the dual-scale channel attention module, and the following steps are performed as follows:

(1) Passing through the encoder.

To capture the attention information between different modalities, the feature map is first sent into the encoder layer of the Transformer [45]. The encoder consists of a self-attention module and a feed-forward neural network. Concretely, the self-attention mechanism is able to interact with the vectors converted from different sequence tokens, providing attention information about the correlation between the different modalities. The basic formula of the self-attention mechanism is first expressed as follows:

(5) $$\begin{array}{c} \left\{\begin{array}{cc} Q & =W_{Q}x\\ K & =W_{K}x\\ V & =W_{V}x \end{array}\right. \end{array}$$

Therefore, the input *x* is converted to a query *Q*, a key *K*, and a value *V* by means of three learnable weights $W_{Q}$, $W_{K}$, and $W_{V}$. Here, *Q* is used to query the similarity of other vectors to itself and *K* is used for indexing for operations.

By dot-multiplying *Q* and *K*, the similarity between the two is computed, which is then converted into a weight probability distribution to obtain the importance of different modalities in different signal sequences as attention information. Specifically, the attention information is normalized via scaling factor and softmax.

(6) $$\begin{array}{c} Atten\left(Q,K,V\right)=softmax\left(\frac{QK_{T}}{\sqrt{d_{k}}}\right)V \end{array}$$

Finally, the output of this self-attention layer is obtained by weighting the value *V*, which helps in the classification with the attention information and then accumulating it. Utilizing multiple self-attention layer operations, the multi-head attention layer is as shown in the following equation:

(7) $$\begin{array}{cc} G(X)=Multihead(Q,K,V) & =concat(head_{1},head_{2},head_{n})W \end{array}$$

(8) $$\begin{array}{cc} head_{n} & =Atten\left(Q_{n},K_{n},V_{n}\right)s_{n}ftmax\left(\frac{Q_{n}K_{n}^{T}}{\sqrt{d_{k}}}\right)V_{n} \end{array}$$

(2) Construct the global channel attention matrix.

First, feature mappings across spatial dimensions H×W are aggregated after a squeeze compression operation. A channel descriptor containing global attention information is generated via global average pooling, which is denoted as follows:

(9) $$\begin{array}{c} z_{i}=F_{squ}\left(x_{i}\right)=\frac{1}{H\times W}\sum_{m=1}^{H}\sum_{n=1}^{H}x_{i}\left(m,n\right) \end{array}$$

After squeeze compression, the aggregated information is sent into two convolution layers to capture the channel dependencies.

(10) $$\begin{array}{c} L\left(X\right)=F_{conv}\left(z\right)=B\left(Conv_{1}\left(\sigma \left(B\left(Conv_{2}\left(z\right)\right)\right)\right)\right) \end{array}$$

where σ and *B* represent the Rectified Linear Unit (ReLU) function and Batch Normalization (BN), respectively. Specifically, the Conv that we used is point-wise convolution, which enhances the nonlinear capabilities of the network. The kernel sizes of $Conv_{1}$ and $Conv_{1}$ are 1×1×1 and 3×1×1, respectively.

(3) Matrix multiplication between the attention matrix and the original features.

(11) $$\begin{array}{c} H^{\prime }=H⊗W(\left(X⊕Y\right)=H⊗\sigma \left(L\left(X\right)⊕G\left(X\right)\right), \end{array}$$

where ⊗ represents matrix addition and ⊕ is matrix multiplication. W(X) contains the summation information of local attention L(X) and global attention G(X), extracted through DCAM.

#### 3.2.3. Iterative Dual-Channel Attention Module (iDCAM)

The inputs are high-level feature maps *X* and low-level feature maps *Y*. *X* utilizes the local sensing and context-sensitive inference capabilities of CNN and LSTM to capture the discriminative properties of each modality. However, the extracted high-level features are rich in local semantic information but ignore inter-modal difference information. In contrast, *Y* extracts global information with a larger perceptual field, and the extracted low-level features extract the distinctiveness between different modalities from a holistic perspective. However, due to the use of fewer convolutional layers, the deep feature semantic information is difficult to mine. Therefore, due to the desire to complement the advantages of low-level features and high-level features, the iDCAM is designed.

**Figure 4:**
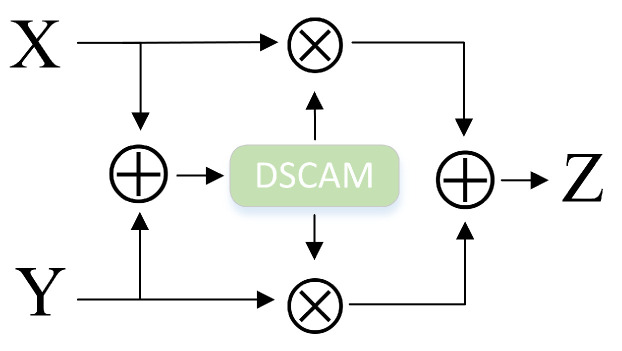
The iterative attention mechanism, iDCAM.

When a layer of DCAM is used, the feature maps X and Y are balanced and weighted to output the first attentional weight like W(X⊕Y)⊗X+(1−W(X⊕Y)⊗X, as shown in Figure 4.

**Figure 5:**
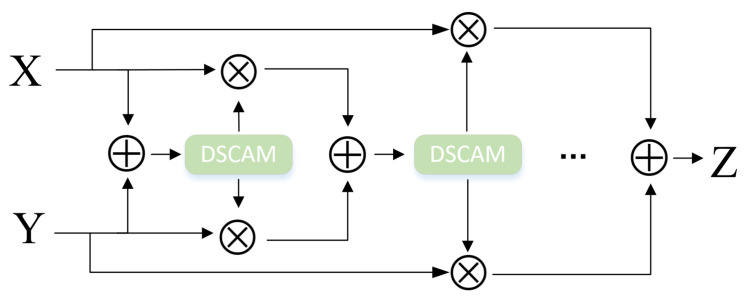
The iterative attention mechanism, iDCAM.

By stacking the DCAM designed in the previous section, iDCAM assigns multimodal attention weights to different modality features.

(12) $$\begin{array}{c} Z_{i}=W_{i}\left(X⊕Y\right)⊗X+(1-W_{i}\left(X⊕Y\right)⊗X,i=1,2,\ldots k \end{array}$$

where W(X⊕Y) represents the summation information of local *X* and global *Y* (Figure 5).

To further clarify the algorithm process, the pseudo-code of the whole training of our method is shown below (Algorithm 1):
**Algorithm 1** IDAF**Input:** 
Initial data**Output:** 
Predicted modulation labels 1:**Data Processing:** Process the initial data into $V_{IQ},V_{AP},V_{SA}$ 2:**Data embedding: **$X\leftarrow x_{m}=E_{x}\left(h_{m},\omega_{m}^{E_{x}}\right),$$Y\leftarrow y_{m}=E_{y}\left(h_{m},\omega_{m}^{E_{y}}\right),\left(m\in \left(IQ,AP,SA\right)\right)$Map the processed data through the local and global embedding layer $E_{x}$ and $E_{y}$. 3:**Weighting: for** i=1,2,3…, **do**:**DCAM:** $Z_{i}=W_{i}\left(X⊕Y\right)⊗X+(1-W_{i}\left(X⊕Y\right)⊗X,i=1,2,\ldots k$ 4:**end for** 5:**Cross-self-attention Encoder:**Predicted labels y← Encoder(X, Z) 6:**return** Predicted labels *y*.

#### 3.2.4. Cross-Self-Attention Encoder

After passing through iDCAM, features are sent into the decoder, along with the sum of intermediate features. The features are fed into the decoder after being assigned weights via iDCAM, and decoding is guided by a cross-attention mechanism, using the sum of the intermediate features $x_{m}$ (m∈(IQ,AP,SA)) and $y_{m}$ (m∈(IQ,AP,SA)).

## 4. Experiment Results and Discussion

### 4.1. Datasets and Implemented Details

#### 4.1.1. Datasets and Implemented Details

The RadioML2016.10a dataset is used as a baseline for training and evaluating the performance of the proposed model. The dataset was created using the GNU Radio [R1] synthetic dataset with commercially available modulation parameters. This dataset also includes many practical channel defects such as channel frequency offsets, sample rate offsets, and additive Gaussian white noise along multipath fading. The experiments are performed on the publicly available dataset RadioML2016.10a benchmark dataset, which consists of 11 modulated signals with signal-to-noise ratios ranging from −20 to 18 dB at 2 dB intervals, including BPSK, QPSK, 8PSK, 16QAM, 64QAM, BFSK, CPFSK, PAM4, WB-FM, AM-SSB, and AM-DSB. it contains 4 samples/symbol (sps) of the modulated signal, with a sample length of 128 samples. This dataset extracts 128 samples per step and offsets 64 samples, so that the size of each signal data is 2 × 128. A complete list of the parameters of the modulation dataset used and the details of the generation can be found in Table 3. Meanwhile, we use a larger dataset RML2016.10B with 1.2 million samples to validate the algorithm’s generalization and pervasiveness. The dataset has 10 modulations: 8PSK, BPSK, CPFSK, GFSK, PAM4, 16QAM, 64QAM, QPSK, AM-DSB, and WBFM.

In our experiments, the dataset is divided into the training set, testing set, and validation set in the ratio of 6:2:2. The training and prediction of our iDAF model and other mainstream models are replicated on an Nvidia Tesla V100 in a Pytorch deep learning algorithm platform. The optimizer used is Adam. The learning rate is set to 0.0001 and this decreases in an orderly manner following an exponential decay.

#### 4.1.2. Evaluation Metrics

**Figure 6:**
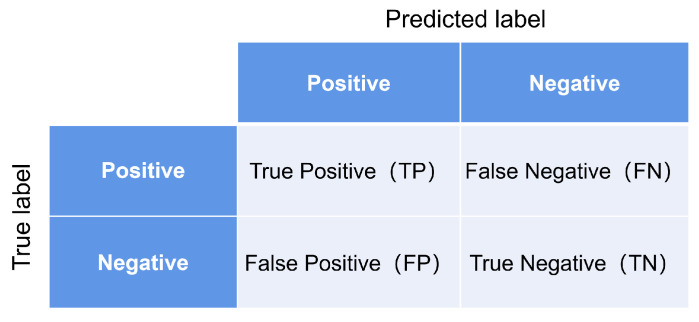
The confusion matrix of binary classification.

Similar to most of the existing methods, the evaluation metric that we adopt is accuracy, which is widely used in image classification tasks, as well as the top-1 accuracy rate and the F1 score. The accuracy rate is proposed based on the confusion matrix of the binary classification task, as shown in Figure 6.

In order to provide a more intuitive picture of the statistical characteristics of classification performance, confusion matrices are often used to observe classification effects. In the confusion matrix, each column represents the predicted value and each row represents the true value. Thus, each element in the confusion matrix represents the number of times that a sample was predicted for a particular category. As shown in the figure, TP (True Positive) indicates that the actual is a positive case and the prediction is positive, i.e., the prediction is correct, and the same can be obtained for TN (True Negative), FP (False Positive) and FN (False Negative). Therefore, the formula for Accuracy can be expressed as:

(13) $$\begin{array}{c} Accuracy=\frac{TP+TN}{TP+TN+FP+FN} \end{array}$$

The numerator (TP+TN) represents the number of correctly predicted samples and the denominator (TP+TN+FP+FN) represents the total number of samples; the accuracy rate can reflect the overall prediction ability of the algorithm model. Further, Top-1 Accuracy can be interpreted as whether the input image is consistent with the real category in the category with the highest probability of predicted output.

The precision indicates the number of actual positive samples out of those predicted to be positive, which is expressed as:

(14) $$\begin{array}{cc} precision & =\frac{TP}{TP+FP} \end{array}$$

Recall indicates the proportion of actual positive samples that are judged to be positive.

(15) $$\begin{array}{cc} recall & =\frac{TP}{TP+FN} \end{array}$$

Combining precision and recall, a heavily used evaluation metric in deep learning is the F1 score (F1-Score), which is usually defined as:

(16) $$\begin{array}{c} F1score=2\frac{precision\times recall}{precision+recall} \end{array}$$

When targeting the modulation recognition task for the eleven classifications in this paper, we use F1 against a single modulation style as an evaluation metric, defined as:

(17) $$\begin{array}{c} F1_{m}=\frac{2c_{m}}{p_{m}+q_{m}} \end{array}$$

where the number of samples correctly identified as modulation m is $c_{m}$, the number of samples identified as modulation m is $p_{m}$, and the number of modulation m samples is $q_{m}$.

### 4.2. Comparative Validity Experiments

#### 4.2.1. Comparison with Uni-Modal and Other AMR Networks

**Figure 7:**
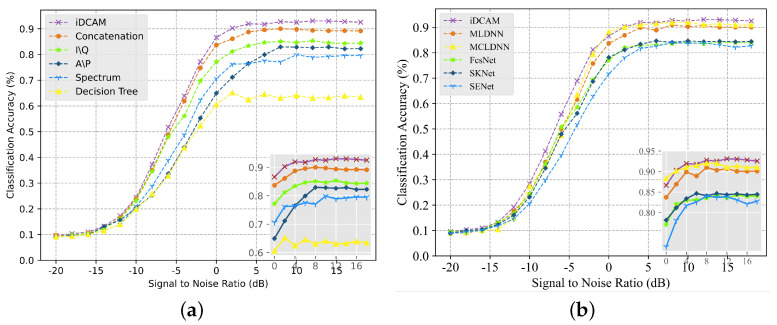
The accuracy plot of the comparison. (**a**) Comparison with uni-modal DL-AMR and FB-AMR methods. (**b**) Comparison with multi-modal DL-AMR and dual-channel attention methods.

**Figure 8:**
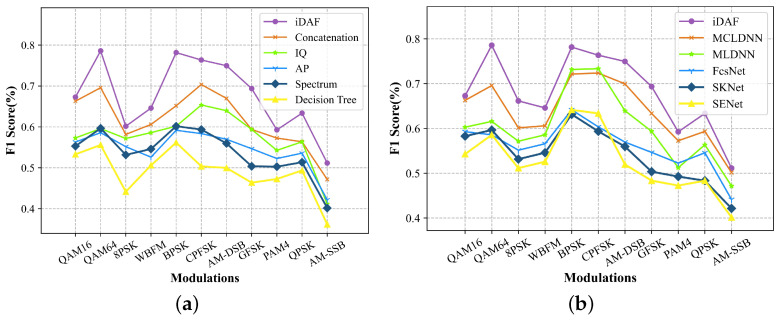
The F1 score plot of the comparison. (**a**) Comparisons of feature extraction models with local embedding layer. (**b**) Comparison with multi-modal DL-AMR and dual-channel attention methods.

To validate the effect of the local embedding layer, we conducted experiments to compare the recognition capabilities at several classical networks that extract features from the signal data. Figure 7 and Figure 8 illustrate the trend of recognition accuracy and F1 score, with SNR from −20 to 18 dB for different models. As can be seen from the figure, the classical FB-AMR model Decision tree and the uni-model methods with IQ, AP, and SP are unable to accurately extract signal features due to the lack of adaptation to the signal data. Without residual concatenation of the original data and the long-term features extracted by the CNN, the resulting feature map is not a holistic view that fuses multimodal long-term and short-term information. The highest accuracy amount of 93.5% is achieved at 10 dB for iDCAM, and the recognition accuracy is 0.6232 at full SNR.

#### 4.2.2. Comparison of iDCAM and Other Attention Mechanisms

In order to assign different weights to the features of different modalities, the attention layer not only needs to extract local high-level semantic information, but also pay attention to the global attention of the channel. For the current attention methods, channel attention mechanism SENet [26], combined spatial attention mechanism BAM [46] and CBAM [47], multi-branch attentional network SKNet [35], and self-attention mechanism Transformer [45] are compared to verify the superiority of the proposed iDCAM. For signal samples with an initial data shape of 2×128, information in the temporal dimension is not sufficient for recognition, but combining channel attention can effectively extract features. As shown in Table 4, SENet clearly outperforms SKNet when used alone, and CBAM with fused channel and spatial attention has a better recognition accuracy. As for the parameters that require calculation iDCAM requires only a small number of parameters for accurate recognition, without relying on a backbone feature extraction network such as Resnet.

#### 4.2.3. Comparison with State-of-Art DL-AMR Methods

In order to verify the effectiveness of the proposed multimodal approach, the effects of the IQ, AP, and SP uni-modal inputs and multiple multimodal approaches are compared under the same experimental setup, respectively.

**Figure 9:**
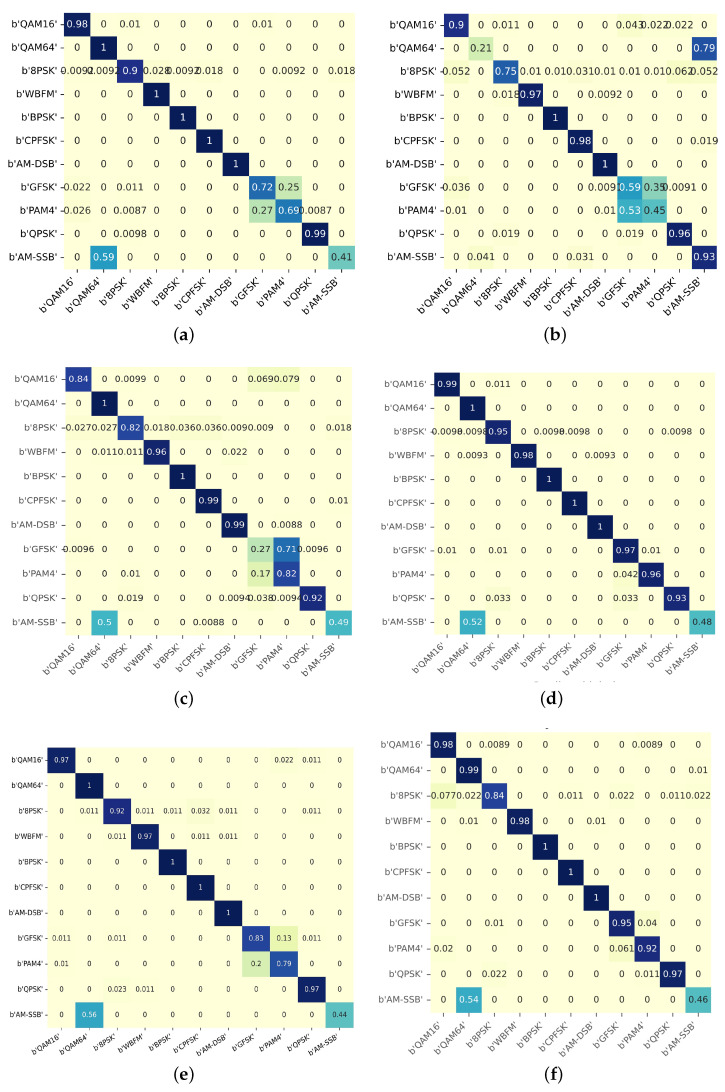
Therecognition performances of different modes to 11 modulations. Darker colours represent a higher probability of current recognition occurring and vice versa. (**a**) CLDNN [15] model with IQ input. (**b**) LSTM [16] model with AP input. (**c**) CNN model with spectrum input. (**d**) iDCAM model with multiple inputs. (**e**) FcsNet model with multiple inputs. (**f**) CBAM model with multiple inputs.

Firstly, from the comparison of single-modal confusion matrices in Figure 9a–c, it can be seen that the model effect of a single-modal input without iDCAM performs poorly on specific modulations. For example, the accuracy of IQ and SP on AM-SSB is less than 50%, and AP is prone to misclassify QAM64 as AM-SSB. After fusing multimodal features through our attention mechanism iDCAM in Figure 9d, the strengths of different modalities compensate for each other and greatly improve the shortcomings.

Secondly, the comparison with other multimodal and dual-channel attentional methods also validates the effectiveness of iDCAM. As shown in Figure 7b and Figure 8b, iDCAM leads most of the methods, and the recognition accuracy at full signal-to-noise ratio exceeds that of the MCLDNN [48] method by 1.5%. We attribute this to iDCAM’s ability to adaptively assign local-global attention weights to multimodal features, as opposed to other methods that directly connect or cross-connect features in a fixed order.

In addition to RML2016.10A, we used a larger dataset RML2016.10B with 1.2 million samples to validate the algorithm’s generalization and pervasiveness over other state-of-art DL-AMR methods. As shown in Table 5 and Table 6, the proposed iDAF method demonstrates outstanding performance over other state-of-art multimodal fusion methods in terms of top1-acc, F1 scores, number of parameters, and training on both public datasets.

### 4.3. Ablation Studies

#### 4.3.1. Ablation Experiments at Different Scales with DCAM

First, single-scale comparisons are performed to verify the superiority of the dual scale. For our proposed dual-scale approach, local scale and global scale extract attentional information from different receptive fields. As shown in Table 7, a single scale leads to the loss of local details or global attention when designing the attention extractor with only local or global branches. The local branch is about 1% more accurate than the global branch. This indicates that the local structure focuses on high-level features and ignores the specificities of different modality data, while the global structure fails to achieve accurate recognition due to a lack of feature details.

**Figure 10:**
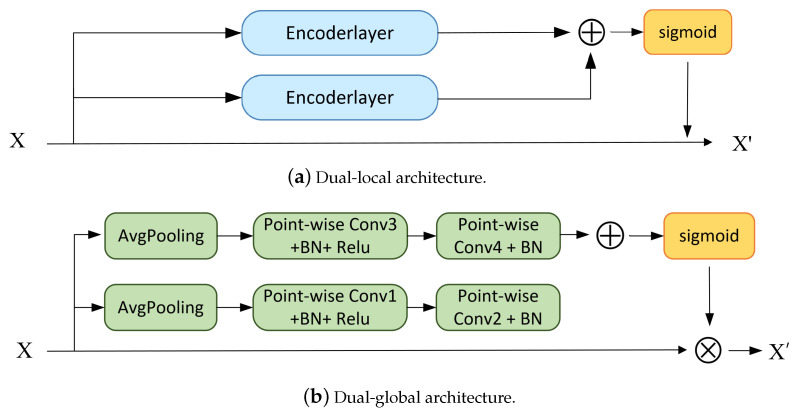
Comparison with different dual structures.

Second, ablation experiments were performed at different dual scales. Specifically, we replicated the individual local or global attention mechanisms separately to compose a dual-local and dual-global structure. As shown in Figure 10, dual-local and dual-global represent that both branches are set to the same receptive field. From the results of the ablation experiments in Table 7, it seems that the dual-branch structure is generally better than the single-branch ones. Model recognition works best only when both the local and global information are fused attentively, while the amount of calculations is relatively reasonable. The two-branch structure increases the FLOPs compared to the single branch, but it maintains a controlled growth rather than an unacceptable one.

#### 4.3.2. Ablation Experiments with Iterative Layers of iDCAM

In order to obtain the number of iteration layers for optimal recognition results of iDCAM, one, two, three, and four iterations of iDCAM are used for modulation recognition, respectively. As shown in Table 8, the best results can be achieved with two iterations. When DCAM is not iterated (i.e., the number of layers is one), attentional information extraction is not adequately extracted. In contrast, the deep network leads to a decrease in the correctness rate when the number of layers is too high.

### 4.4. Limitations and Constraints

Although our method has achieved a high level of recognition accuracy, some constraints and limitations will be the focus of the next step of research.

(1) Zero-shot learning (ZSL) scenarios: The attention mechanism may tend to be better at handling a common public dataset, but it may struggle with rare events or classes due to zero-shot learning (ZSL) scenarios. In the practical application of modulation recognition, the modulation is variable and complex, and the prior knowledge of intercepted information is often insufficient; the modulation that needs to be classified is not always known. When unknown modulations appear in the test set, with the existing attention mechanism is, it is difficult to extract effective features from new samples.

(2) Expensive computations:Additionally, self-attention mechanisms require pairwise comparisons between all input elements, which results in a quadratic increase in computation as the input sequence length grows. This can make them computationally expensive, especially for long sequences.

## 5. Conclusions

In this paper, we introduce an iterative dual-scale attentional fusion (iDAF) method to integrate multimodal data. In the proposed method, we realize a significant classification that is superior to the other fusion DL-AMR methods, and we achieve a recognition accuracy of 93.5% at 10 dB and 0.6232 at full SNR. In future work, one promising direction is to further mine the deeper characteristics of different modalities, and to demonstrate the reason for the existence of variability in different modalities by means of mathematical analysis.

## Figures and Tables

**Table 1:** Comparison of input data of different modalities.

Domains	Models	Effects
I/Q	CNN combined with Deep Neural Networks (DNNs) [13], a combined CNN scheme [21]	Achieves high recognition of PAM4 at low signal-to-noise ratio (SNR)
A/P	Long Short Term Memory (LSTM) [16], a LSTM denoising auto-encoder [14]	Well recognize AM-SSB, and distinguish between QAM16 and QAM64 [22]
Spectrum	RSBU-CW with Welch spectrum, square spectrum, and fourth power spectrum [23]; SCNN [18] with the short-time Fourier transform (STFT), a fine-tuned CNN model [17] with smooth pseudo-Wigner–Ville distribution and Born–Jordan distribution	Achieves high accuracy of PSK [23], recognizes OFDM well, which is revealed only in the spectrum domain due to its plentiful sub-carriers [17]

**Table 2:** Summary of dual-channel attention methods.

Name	taskA	taskB
Direct aggregation on X	X+W(Y)⊗Y	SENet [26]
Aggregation after Slicing	$X+W(cat\left[Freq_{y_{1}},Freq_{y_{2}},Freq_{y_{3}}\ldots \right])⊗Y$	FcaNet [32]
Direct aggregation on Y	W(x)⊗X+Y	PAN [33]
Gated multiple units	F(GMU(BAN(X,Y;A)))	DABERT [34]
Balanced weighting	W(X+Y)⊗X+(1−W(X+Y))⊗Y	SKNet [35]
Iterative balanced weighting	$W_{i}\left(X⊕Y\right)⊗X+(1-W_{i}\left(X⊕Y\right)⊗X$	iDAF

**Table 3:** Comparison of input data of different modalities.

Dataset Content	Parameter Information
Software platform	GNUradio+Python
Data type and shape	I/Q (in-phase/orthogonal), 2 × 128
Modulations	8 digital modulations: 8PSK, BPSK, CPFSK, GFSK, PAM4, 16QAM, 64QAM, QPSK; 3 analog modulations: AM-DSB, AM-SSB, WBFM
Sample size	Each modulation has 2000 signal samples for a total of 220,000
Signal-to-noise ratio	2dB intervals from −20 dB to 18 dB
Channel environment	Additive White Gaussian Noise, Sample Rate Offset (SRO), Rician, Rayleigh, Center Frequency Offset (CFO)
Sample rate	200 kHz
Sample rate offset standard deviation	0.01 Hz

**Table 4:** Comparison of multiple attention mechanisms.

Model	Accuracy	Params (M)
SENet-ResNet18	0.6032	11.9
SKNet-50	0.5994	27.6
CBAM-ResNeXt50	0.6082	27.8
Self-attention	0.618	63.5
BAM-Resnet-50	0.6038	24.7
FcsNet	0.6069	37.4
iDCAM	0.6232	6.9

**Table 5:** Comparison of state-of-the-art DL-AMR methods on the RML2016.10A dataset.

Model	Accuracy	Top1-Acc (Average)	F1 Score (Average)	FLOPS	Train Epochs
GRU	0.5374	72.9%	56.3%	89,531	10
DAE	0.5632	75.7%	59.8%	67,682	9
CLDNN	0.5982	76.3%	61.1%	0.7 G	11
MCLDNN	0.618	79.4%	64.2%	8.4 G	21
HKDD [49]	0.6094	77.6%	62.7%	21.7 G	38
MLDNN [11]	0.6106	78.5%	63.2%	36.7 G	45
iDAF	0.6232	80.5%	65.4%	10.9 G	34

**Table 6:** Comparison of state-of-the-art DL-AMR methods on the RML2016.10B dataset.

Model	Accuracy	Top1-Acc (Average)	F1 (Average)
GRU	0.5732	75.3%	60.3%
DAE	0.5994	76.2%	62.2%
CLDNN	0.6082	77.1%	62.6%
MCLDNN	0.6314	80.8%	65.9%
HKDD	0.6198	78.2%	64.1%
MLDNN	0.6226	80.4%	64.8%
iDCAM	0.6483	81.2%	66.7%

**Table 7:** The ablation results of different scales.

Architectures	Recognition Accuracy	FLOPs (G)
Local	0.618	10.1
Global	0.6081	/
Dual-local	0.6192	20.2
Dual-global	0.6104	/
Local-global	0.6232	10.9

**Table 8:** The ablation results of iterative layers.

Iterations K	One-Layer	Two-Layer	Three-Layer	Four-Layer
**Accuracy**	0.6194	0.6232	0.6204	0.6181

## Data Availability

Not applicable.
